# A Comprehensive Survey on Visual Perception Methods for Intelligent Inspection of High Dam Hubs

**DOI:** 10.3390/s24165246

**Published:** 2024-08-14

**Authors:** Zhangjun Peng, Li Li, Daoguang Liu, Shuai Zhou, Zhigui Liu

**Affiliations:** 1 School of Information Engineering, Southwest University of Science and Technology, Mianyang 621010, China; pzj1@swust.edu.cn (Z.P.); liudaoguang@mails.swust.edu.cn (D.L.); handsome@mails.swust.edu.cn (S.Z.); 2 School of Computer Science and Technology, Southwest University of Science and Technology, Mianyang 621010, China; ll@swust.edu.cn; 3 Sichuan Engineering Technology Research Center of Industrial Self-Supporting and Artificial Intelligence, Mianyang 621010, China

**Keywords:** high dams, safety inspection, defects identification, environmental perception, image enhancement, defects quantitative analysis, deep learning

## Abstract

There are many high dam hubs in the world, and the regular inspection of high dams is a critical task for ensuring their safe operation. Traditional manual inspection methods pose challenges related to the complexity of the on-site environment, the heavy inspection workload, and the difficulty in manually observing inspection points, which often result in low efficiency and errors related to the influence of subjective factors. Therefore, the introduction of intelligent inspection technology in this context is urgently necessary. With the development of UAVs, computer vision, artificial intelligence, and other technologies, the intelligent inspection of high dams based on visual perception has become possible, and related research has received extensive attention. This article summarizes the contents of high dam safety inspections and reviews recent studies on visual perception techniques in the context of intelligent inspections. First, this article categorizes image enhancement methods into those based on histogram equalization, Retinex, and deep learning. Representative methods and their characteristics are elaborated for each category, and the associated development trends are analyzed. Second, this article systematically enumerates the principal achievements of defect and obstacle perception methods, focusing on those based on traditional image processing and machine learning approaches, and outlines the main techniques and characteristics. Additionally, this article analyzes the principal methods for damage quantification based on visual perception. Finally, the major issues related to applying visual perception techniques for the intelligent safety inspection of high dams are summarized and future research directions are proposed.

## 1. Introduction

High dams serve as vital strategic infrastructure, bolstering the efficient development of hydropower and the comprehensive utilization of water resources. They continually and stably fuel economic and social development by providing energy supply and flood protection, thus generating substantial social and economic benefits. The safety of high dams is a critical component of national public safety, with the assurance of their secure operation and the full realization of their functions being a strategic necessity. The routine inspection of high dams has become an essential measure to guarantee their safe operation. In recent years, with the rapid advancement of unmanned equipment, computer vision, machine learning, and intelligent perception technologies, among others, widespread research and applications have been conducted in the field of construction safety monitoring and inspection techniques based on visual perception.

In the domain of construction safety monitoring, Ye et al. [1] briefly summarized the application of image processing techniques in safety inspections and reviewed the potential error sources in vision perception methods. Meanwhile, Feng et al. [2] outlined the displacement measurement methods based on visual perception from both hardware and software perspectives, and reviewed the extended applications related to construction safety patrol tasks that utilize the obtained displacement data. Furthermore, Xu et al. [3] conducted a detailed review of the development of displacement measurement algorithms based on visual perception, summarizing the applications developed on the basis of vision-based displacement measurements. Spencer et al. [4] summarized advancements in the field of structural inspection and monitoring based on visual perception, presenting methods for incorporating deep learning-based visual perception techniques in structural engineering applications. They also discussed the applications of static and dynamic displacement measurements based on visual perception in laboratory and field scenarios. Dong et al. [5] analyzed and summarized the application and development of computer vision technology in the context of local and global structural health monitoring.

The above literature has outlined both the mainstream methods and recent advancements in visual perception technology for construction safety monitoring and inspection. However, comprehensive reviews on visual perception techniques and methods for use in the intelligent safety inspection of high dams remain scarce. In recent years, researchers have introduced numerous visual perception methods tailored for the inspection of specific building structures and, despite the scarcity of studies focusing on high dams in this regard, methods designed for similar characteristic structures hold significant reference value. This article serves to primarily review visual perception methodologies for the intelligent inspection of high dams and analogous structures, highlighting related research findings and advancements. Furthermore, it anticipates future technological development trajectories in this domain. This article primarily synthesizes the latest achievements and developments in visual perception methods and key application technologies for intelligent safety inspections of high dams, allowing the direction of technological advancements to be anticipated.

## 2. High Dam Safety Intelligent Inspection Based on Visual Perception

### 2.1. High Dam Safety Inspection

In accordance with the ‘Dam Safety Management: Pre-operational Phases of the Dam Life Cycle’ (International Commission on Large Dams, Bulletin 175, 2021) [6], the ‘Regulation on the Administration of Reservoir Dam Safety’ (Decree No.77 of the State Council of the People’s Republic of China, 1991) [7], and other provisions, regular safety monitoring and inspections need to be carried out on the hydraulic buildings of hydropower hubs during the operation period.

**Figure 1:**
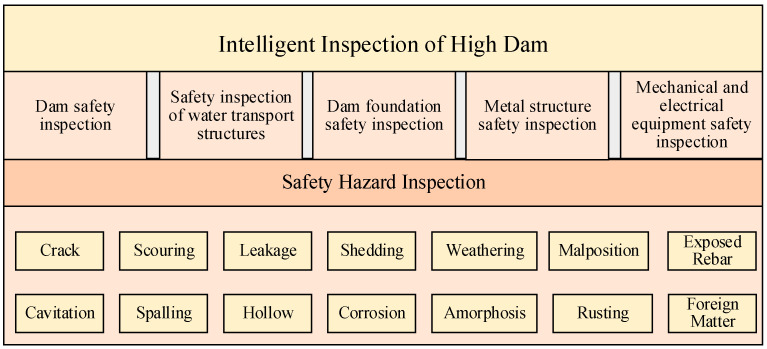
Overview of high dam intelligent safety inspection.

Based on the safety patrol inspection analysis detailed in the ‘Guidelines on the Patrol and Inspection of Reservoir Dam Safety’ (Dam Safety Management Center of the Ministry of Water Resources, China, ISBN: 9787517094777, 2021) [8] and ‘Federal Guidelines for Dam Safety’ (Department of Homeland Security, USA, FEMA P-93, 2023) [9], the primary goal of safety inspections for high dam hubs is to identify potential safety hazards in the structures of the high dam hubs. Typical safety hazards in the structures of high dam hubs include defects such as concrete cracks, cavitation, erosion, spalling, weathering, hollow drumming, falling off, corrosion-induced coarse aggregate exposure, pits, troughs, exposure of reinforcing steel, metal corrosion, deformation, displacement, seepage, and foreign objects. During the inspection process, it is necessary to identify the location, scale, and shape of the defects, as detailed in Figure 1. Most of these safety hazards and their characteristics can be identified through human visual inspection during the safety patrol inspection process. Therefore, visual perception methods can be utilized to achieve intelligent inspections.

### 2.2. The Application of Visual Perception in Intelligent Inspections

**Figure 2:**
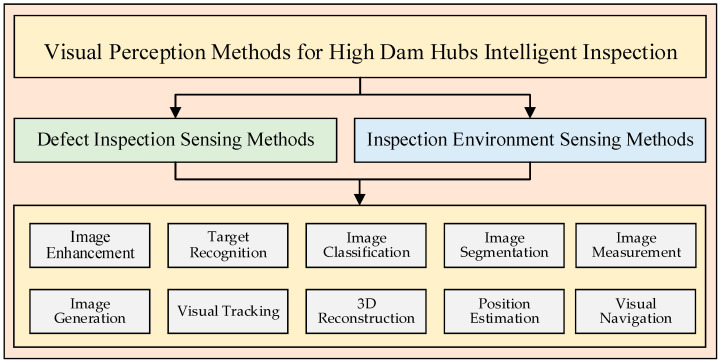
Visual perception methods for high dam intelligent safety inspection.

In intelligent safety inspections, visual technologies such as image classification, measurement, detection, recognition, and segmentation based on visual perception are utilized. These technologies can extract local structural health safety state indicators from visible images of a structure’s surface, including cracks, spalling, corrosion, delamination, and voids. Using methods such as optical flow and visual tracking, global health safety state indicators of high dam structures, such as structural responses including displacement, vibration, deformation, and misalignment, can be obtained. While the tasks and routes of high dam intelligent safety inspections are planned according to prior models and maps, unforeseen situations and temporary tasks outside of the planned factors may occur during the inspection process. Autonomous obstacle recognition and local adaptive inspection route planning based on visual perception are vital means to ensure the reliable completion of intelligent inspections. Visual perception methods can allow for long-distance, non-contact, low-cost, high-efficiency, and automated inspections to be successfully carried out. Figure 2 demonstrates the relevant visual perception technologies that may be applied in high dam intelligent safety inspections, mainly including image enhancement, target recognition, image classification, image segmentation, image measurement, image generation, visual tracking, three-dimensional reconstruction, position estimation, and visual navigation.

## 3. Visual Perception and Processing of Defects in High Dam

The safety inspection of a high dam requires a thorough analysis of its structural conditions and safety levels through an assessment of defects such as cracks, spalling, delamination, and corrosion. The routine procedure for inspectors is to first visually inspect the structure and then manually mark locations on structural drawings. This process is labor-intensive and time-consuming, requiring a significant workforce. Thus, utilizing new sensors and information processing techniques for defect characteristic perception in high dam safety inspections has aroused widespread attraction in both industry and academia. In recent years, significant advancements have been achieved in this area through the use of computer vision and machine learning methodologies.

**Figure 3:**
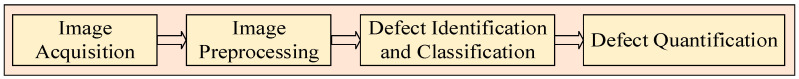
Procedure of the defect perception method.

As illustrated in Figure 3, the procedure for visual defect perception during high dam safety inspections comprises several stages. Initially, image acquisition is conducted, where cameras mounted on intelligent inspection devices capture images of the inspection area. Due to variations in the capture environment, the quality of the collected images may not be uniform. To facilitate efficient recognition, image filtering, and enhancement procedures must be carried out. Then, the processed high-quality images can be used for defect identification and classification tasks and, ultimately, defect parameters are quantified to furnish supporting data for subsequent safety evaluations.

### 3.1. Image Enhancement Methods

**Figure 4:**
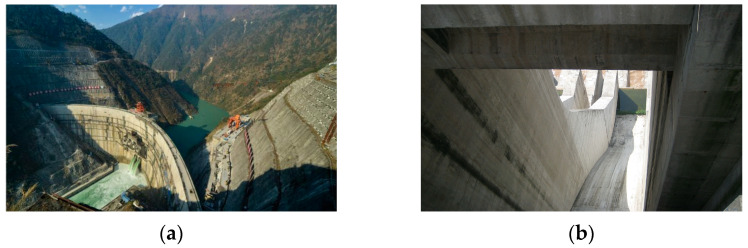
Image under the influence of the high dam environment: (**a**) uneven illumination image of the high dam; and (**b**) low-light image of the high dam.

The quality of images and videos greatly influences the accuracy of visual perception systems. Many high dams are situated in mountainous regions with complex environments and intricate structural objects to inspect, which are easily affected by lighting, rain, snow, and other factors in fluid–solid environments. This leads to issues such as substantial background noise, loss of detail, uneven illumination, low light, and decreased resolution of images, as illustrated in Figure 4. Therefore, image enhancement is required before performing high-dam structural defect perception.

The main purposes of high dam image enhancement are to expand the difference between the features of different objects in the image, suppress the uninteresting features, improve the image quality, enrich the information, strengthen the image interpretation and recognition effect, and meet the requirements of defect and environmental sensing [10]. Image enhancement algorithms can improve the quality of collected inspection images, thus reducing the processing time and increasing recognition accuracy. We analyze the enhancement methods that can be used for high dam images in three categories: histogram equalization (HE) methods, retinex methods, and deep learning methods.

#### 3.1.1. Histogram Equalization Methods for Image Enhancement

The principle of histogram equalization involves evenly distributing the grayscale values in an original image from a relatively concentrated range across the entire grayscale space. This process achieves nonlinear stretching of the image and redistributes its pixel values, thereby enhancing the image’s contrast and dynamic range through the analysis and redistribution of pixel grayscale values based on histogram distributions [11]. For example, Wang et al. [12] proposed the utilization of the adaptive histogram equalization (AHE) method to compute local histograms, subsequently redistributing brightness levels to enhance an image’s contrast while preserving a significant amount of detail. However, this approach inherently increases the time complexity by segmenting the image into multiple distinct sections. Additionally, Wang et al. [13] presented a Weighted Threshold Histogram Equalization (WTHE) algorithm, which is suitable for video enhancement, effectively mitigating issues related to over-enhancement and average saturation artifacts, despite the algorithm’s susceptibility to high noise levels and incomplete detail preservation. Lee et al. [14] employed the Local Difference Representation (LDR) method to amplify the differential representation of adjacent pixels in grayscale within a two-dimensional histogram, demonstrating not only superior speed but also enhanced efficacy. Moreover, Li et al. [15] introduced an underwater image enhancement technique based on the underwater dark channel model, which minimizes information loss and relies on prior knowledge of the histogram distribution for image de-fogging. Nonetheless, the method tended to induce over-enhancement, potentially resulting in a loss of image detail. The purpose and merits of each method are listed in Table 1.

#### 3.1.2. Retinex Methods for Image Enhancement

**Figure 5:**
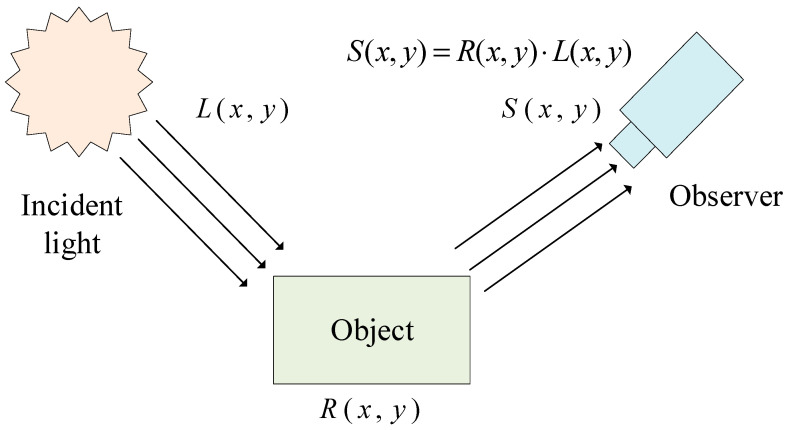
The basic principle of Retinex theory.

Retinex theory, proposed by Land et al. [16] in 1977, involves the decomposition of images into illumination and reflectance components, offering a robust and flexible framework for image enhancement tasks. The core of Retinex theory is that the color of an object is determined by its reflective capacity towards red, green, and blue light, not by the absolute intensity of the reflected light. The color of an object remains consistent regardless of variations in illumination, underscoring the foundation of Retinex theory in color constancy. Based on Land’s theory, a given image S(x,y) can be decomposed into two distinct images: the reflected object image R(x,y) and the incident light image L(x,y). A schematic diagram illustrating this principle is shown in Figure 5.

Stemming from this theory, a range of related methods have been developed, such as the single-scale Retinex (SSR) and multi-scale Retinex (MSR) low-light image enhancement techniques, developed by Jobson et al. [17,18]. The SSR method is sensitive to high-frequency components and enhances edge information in images well, although it might over-enhance the image and lose real information. Compared to SSR, MSR can accomplish color enhancement, color constancy, and local and global dynamic range compression; however, it falls short in terms of smooth edges and improvement of high-frequency detail. Furthermore, Si et al. [19] presented the SSRBF algorithm, which merges SSR and a bilateral filter (BF) to address low and uneven lighting issues, thereby improving video image quality. Xiao et al. [20] presented scaled Retinex with fast mean filtering applied to the luminance component in hue–saturation–value (HSV) color space and proposed a rapid image enhancement method based on color space fusion. Tao et al. [21] presented a fusion framework based on MSR, which integrates Range Covariance Filtering (RCF), Contrast-Limited Adaptive Histogram Equalization (CLAHE), Non-Local Means Filtering (NLF), and Guided Filtering (GF) to correct for uneven illumination and remove noise in images. Gu et al. [22] suggested an enhancement for severely underlit images based on a Retinex model with fractional-order variation, while Hao et al. [23] advanced the field by introducing a semi-decoupled decomposition-based method for low-light image enhancement. Moreover, Zhang et al. [24] utilized a bidirectional perceptual similarity method to enhance underexposed images. These approaches transform the Retinex approach into a statistical inference problem, solving image enhancement challenges through the application of various constraints with good results. However, when considering images characterized by a complex environment, such as those commonly observed in high dam inspection scenarios, these algorithms still have the problems of low distortion and reduction. The purpose and merits of each method are listed in Table 2.

#### 3.1.3. Deep Learning Methods for Image Enhancement

Traditional image enhancement methods often face problems after adjusting the color, brightness, and contrast of the image, such as amplified noise, loss of details, and color distortion. With the advancement of deep learning approaches, they have recently been widely applied in image enhancement tasks by many scholars. Image enhancement methods for low-light images based on deep learning are data-driven approaches in which the model autonomously learns the features of images under standard lighting conditions. This method aims to diminish the impact of light on the image, enhancing its overall quality.

The prevalent structure for image enhancement algorithms based on deep learning is the encoder–decoder architecture. LLNet [25] stands out as the pioneering algorithm for enhancing low-light images through deep learning, achieving remarkable outcomes. In LLNet, a modified version of the stacked-sparse denoising autoencoder is utilized to learn from synthetically darkened and noise-injected training samples, effectively enhancing images captured in natural low-light settings or those affected by hardware degradation. Ren et al. [26] aimed to improve the visibility of low-light images using a trainable hybrid network, which incorporates encoder–decoder architecture to estimate global content, while introducing a novel spatially variant recurrent neural network (RNN) as an edge stream to model edge details. Tao et al. [27] proposed a convolutional neural network (CNN)-based model to denoise low-light images with a bright channel prior to estimating the transmission parameter. Some scholars have combined the concept of Retinex with deep learning algorithms such as CNNs to conduct research on their image enhancement effect. For example, Li et al. [28] introduced a method for low-light image enhancement based on a constrained low-rank approximation Retinex model. Meanwhile, Cai et al. [29] introduced an enhancement algorithm based on Retinex and deep learning, replacing the CNN with a weighted least squares method for image decomposition. Li et al. [30] presented LightenNet, which employs a CNN to generate illumination maps and traditional methods for enhancement. Retinex-Net [31] has been presented by Wei et al., which consists of a Decom-Net for splitting the input image into lighting-independent reflectance and structure-aware smooth illumination and an Enhance-Net for illumination adjustment. These methods fully combine the advantages of Retinex and CNN, obtaining better enhancement effects for specific scenes.

Some scholars have paid attention to the role of Generative Adversarial Networks (GANs) in image enhancement. Shi et al. [32] proposed Retinex-GAN, an early attempt at combining Retinex with GANs. Yang et al. [33] proposed a method for enhancing low-light images through adversarial training using both paired and unpaired data. Notably, all of the above methods were trained only on paired data, while not utilizing a vast amount of unpaired data. Addressing this gap, Chen et al. [34] and Jiang et al. [35] subsequently introduced the Deep Photo and EnlightenGAN methods, respectively, capitalizing on unpaired data for training to achieve image enhancement. Meanwhile, there have been many recent studies on deep learning-based image enhancement. For instance, Wang et al. [36] introduced a simple low-light image enhancement model based on the Weber–Fechner law in log space. Lu et al. [37] used gradient prior-assisted networks for low-light image enhancement, while Rasheed et al. [38] focused on a brightness super-resolution deep network using super-resolution methods. Zhou et al. [39] presented a novel multi-feature underwater image enhancement method with an embedded fusion mechanism (MFEF). Considering that underwater images usually have the characteristics of low contrast, color distortion, and blurred details, WB and CLAHE input processing methods were utilized to obtain superior quality inputs, and the MFF module was used to make the multiple forms of features fully interactive while retaining a great level of detail. This allows valuable features to be highlighted while the unhelpful features are inhibited using PCAM. Overall, methods considering multiple colors have great practical value for high-dam image enhancement.

Some researchers have recently paid attention to the value of transformers for image enhancement. Wang et al. [40] presented LLFormer, a transformer-based low-light enhancement method. The core components of LLFormer are the axis-based multi-head self-attention and cross-layer attention fusion block, which significantly reduce the linear complexity of the model. Cai et al. [41] proposed a novel transformer-based method, Retinexformer, for low-light image enhancement. Through analyzing the corruptions hidden in under-exposed scenes caused by the brightening process, perturbation terms were introduced into the original Retinex model to formulate the one-stage, Retinex-based framework (ORF). Then, they designed an Illumination-Guided Transformer (IGT) that utilizes the illumination information captured by the ORF to direct the modeling of long-range dependencies and interactions between regions with different lighting conditions. The Retinexformer was derived by integrating the IGT and ORF. In general, image enhancement methods combined with transformers provide better performance. The performance metrics for the deep learning methods used for image enhancement detailed above are listed in Table 3.

**Figure 6:**
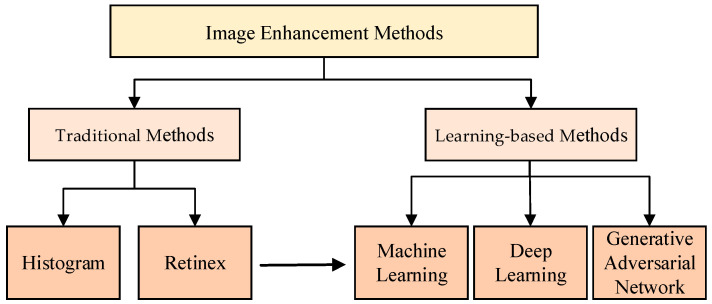
The research trends for image enhancement methods.

From the analysis of the aforementioned image enhancement methods, the trends in image enhancement research are depicted in Figure 6.

### 3.2. Visual Perception Methods for Concrete Defects

Researchers have recently introduced a plethora of vision-based methods for the perception of specific structural defects in concrete. However, there is a noticeable gap in research concerning concrete structures in the high dam setting, underscoring the significance of similar methodologies for reference. In this section, we delve into research findings pertaining to concrete crack detection, the identification of other types of defects, and defect quantification.

#### 3.2.1. Visual Perception Methods for Concrete Cracks

**Figure 7:**
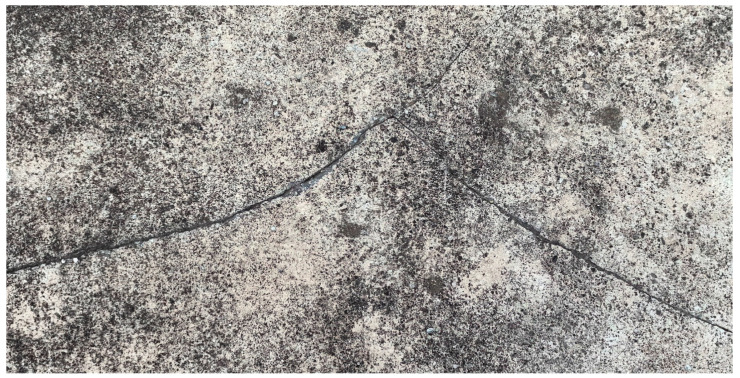
Picture of cracks in the concrete surface of a high dam.

Most structural defects evolve from cracks, with many types of internal damage manifesting in the form of cracks. An image of cracks on the concrete surface of a high dam is shown in Figure 7. The original images collected during inspections have complex backgrounds, making detection and quantification challenging. There have been numerous recent studies on visual perception methods for crack detection, targeting structures such as dams, bridges, roads, tunnels, and so on. At present, visual perception methods for concrete crack detection predominantly hinge on traditional image processing, machine learning, and deep learning methodologies.

(1) Crack Detection Methods based on Traditional Image Processing

In traditional image processing approaches for crack detection, threshold-based methods are used to differentiate between non-crack pixels and pixels within crack areas through the comparison of threshold values, identifying pixels with higher values as cracks. Fujita et al. [42] introduced a threshold-based method for concrete crack detection, which can handle irregular lighting conditions, shadows, and imperfections. However, its sensitivity and adaptability are contingent upon the threshold values. A later improvement by Fujita et al. [43] adopted a probabilistic statistical method for coarse crack localization and a local adaptive threshold technique for fine crack detection, enhancing the effectiveness of identification. Moreover, Talab AMA et al. [44] proposed a floor crack detection method based on the Otsu algorithm, utilizing grayscale images as inputs and applying appropriate threshold values for background and foreground classification. This method leverages Sobel filtering to eliminate residual noise and the Otsu algorithm for crack detection, thereby improving the identification accuracy. Asdrubali et al. [45] developed an algorithm to detect thermal bridge contours using infrared thermal imagery, incorporating the Kantorovich operator to enhance the thermal images. This method analyzes histograms and employs suitable threshold values for the effective detection of detecting contours. Chen et al. [46] developed a method for the detection of concrete cracks based on the OTSU algorithm and differential images. Their experimental results showed that cracks can be discerned from complex backgrounds using their method. These methods primarily focus on utilizing preprocessing, filtering, thresholding, edge detection, and other techniques to enhance the capability of the model to detect and recognize cracks and defects. They have been shown to be effective in specific scenarios, reducing the costs associated with manual labor. Threshold-based methods detect cracks by leveraging the difference in grayscale values between the crack area and its surroundings. However, in high dam inspection images, uneven lighting, noise blur, and shadows make the contrast between crack grayscale values and background regions less distinct, leading to difficulties in crack identification. The merits and limitations of each method are listed in Table 4.

(2) Crack Detection Methods based on Machine Learning

As machine learning algorithms continue to advance, their applications are becoming increasingly widespread across various domains. In recent years, researchers have started to explore the use of machine learning algorithms to address the challenge of perceiving surface cracks in concrete structures, achieving some remarkable results. Machine learning algorithms possess robust data processing and pattern recognition capabilities, enabling them to automatically extract valuable information from large datasets and discover the intrinsic patterns and relationships within the data. This unique capability provides machine learning algorithms with distinct advantages for the perception of surface cracks in concrete structures, enhancing both their accuracy and robustness in perception. For example, Liu et al. [47] employed a support vector machine (SVM) as a classifier to detect cracks in images. This process entailed preprocessing the images to extract features based on pixel intensity, with the SVM training process consistently aiming for global optimization to prevent overfitting. Luo et al. [48] introduced a crack detection method that utilizes a random classifier to distinguish between crack and non-crack images. Fisher et al. [49] presented an SVM-based approach for detecting cracks in embankments using passive seismic data. Fan et al. [50] devised an automated crack detection technique for surfaces of hydropower station dams, leveraging local–global clustering. Nishikawa et al. [51] developed image filters through genetic encoding to eliminate residual noise, proposing a highly resilient method for the identification of surface cracks on concrete structures. Gordan et al. [52] assessed the efficacy of classical edge detection operators in crack detection, proposing a fuzzy C-means clustering edge detection algorithm to aid in crack identification in infrared images.

These methods leverage the powerful capabilities of machine learning algorithms, achieving significant progress in enhancing both their detection accuracy and robustness. Feature extraction methods based on machine learning, similar to those based on traditional image processing, require the manual design of features. Different categories of defects require different types of features for description. Further research is warranted to explore the robustness and adaptability of feature extraction and identification methods for diverse defects in high, concrete dam structures. The merits and limitations of each method are listed in Table 5.

(3) Crack Detection Methods based on Deep Learning

**Figure 8:**
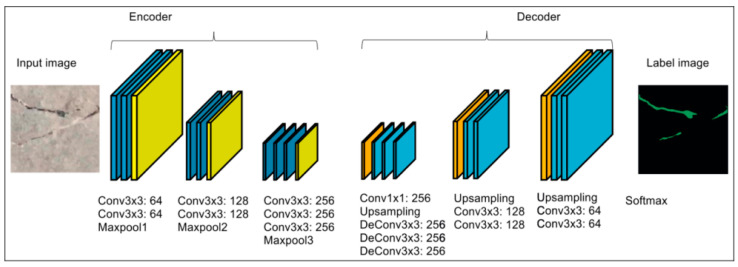
An example of pixel-based crack detection based on FCN.

With the rapid development of deep learning methods in fields such as object classification, recognition, and segmentation, many researchers have begun exploring the development of crack detection methods based on deep learning techniques. Dung et al. [53] trained a Fully Convolutional Network (FCN) model for semantic segmentation to extract cracks from images, providing detailed crack maps with shapes and distributions. Ni et al. [54] combined GoogLeNet with classification and CDN to achieve pixel-level crack detection. In crack detection methods based on deep learning, users only need to input the image into the end-to-end framework to obtain a crack map with detailed information as an output. As shown in Figure 8, a concrete image is input into a pre-trained neural network. After passing through three sets of convolutional layers (encoders) and three sets of deconvolutional layers (decoders), the crack shapes are segmented and displayed as a crack map.

Numerous crack detection methods grounded in deep learning have recently emerged. Feng and Zhang et al. [55,56,57] carried out a series of deep learning-centric investigations concerning defect identification and detection in hydropower station spillway dams. Their research introduced diverse methodologies, including a classification recognition approach for prominent defects, such as cracks in spillway dam structures, leveraging transfer learning and convolutional neural networks. This method effectively identifies defects such as cracks, seepage, and spalling. Additionally, they proposed an automated perception method for pixel-level crack detection on dam surfaces, employing deep convolutional networks, along with a real-time inspection method for flood discharge tunnel defects based on deep learning techniques. Through employing various deep learning networks and optimizing them for spillway dam defect detection, significant advancements were achieved.

Modarres et al. [58] proposed a CNN methodology for crack recognition and classification, utilizing the ReLU activation function to mitigate the gradient explosion problem. Pang et al. [59] developed a technique for detecting visible cracks in hydropower hub facilities by integrating ResNetv2 and Faster-RCNN as a feature extraction network for deep feature extraction. Li et al. [60] suggested a synchronized localization method for concrete crack detection, incorporating crack and geographical information to detect and categorize cracks under challenging conditions without external location data. Deng et al. [61] utilized UAVs to collect images of hydropower station dam surfaces and established a CNN for discerning dam surface cracks. Cheng et al. [62] proposed an enhanced FCN for dam surface crack detection and performed a quantitative crack analysis based on relevant imaging principles.

Zou et al. [63] presented an end-to-end DeepCrack crack detection network architecture, which enables automatic detection through the learning of high-level crack features, further showcasing adaptability to samples with noisy labels. Li et al. [64] proposed an automatic crack classification and localization method for concrete dam structures using deep residual neural networks and transfer learning. Zhu et al. [65] devised an automatic detection and diagnosis method for damages in hydraulic structures, employing the streamlined parameters of the Xception backbone network for efficient crack feature extraction. To combat the challenges of losing details in microcrack images and limited target information in hydraulic concrete structures, they devised an image semantic segmentation algorithm based on Deeplab V3+ and an adaptive attention mechanism. This method, when leveraging a diverse dataset of concrete crack images from hydraulic structures, could effectively identify various crack types across complex background scenarios.

Novel deep learning networks have recently been applied for defect detection on concrete surfaces, achieving good performance. For example, Wu et al. [66] introduced a pixel-level, real-time crack segmentation method based on the LCA-YOLOv8-seg model, proposing a lightweight LCANet backbone network and a lighter prototype mask branch to reduce model complexity. Zhang et al. [67] presented UTCD-Net, a pixel-level crack detection network integrating Transformer and CNN models to capture both local and global crack features, facilitated by an attention fusion module to refine the segmentation crack morphology results. Xiang et al. [68] introduced DTrC-Net, a novel dual-encoder network structure combining the transformer and CNN to simultaneously capture local information and long-range dependencies for crack image segmentation, enhancing the extraction of both global contextual information and local crack features. The merits and selected performance metrics for each method are listed in Table 6.

#### 3.2.2. Visual Perception Methods for Other Defects

There is a diverse range of surface defects of concrete on high dams. This section delves into the analysis of relevant methods and achievements, focusing separately on the detection of multi-type defects and underwater concrete defects.

(1) Detection of Multiple Types of Concrete Defects

**Figure 9:**
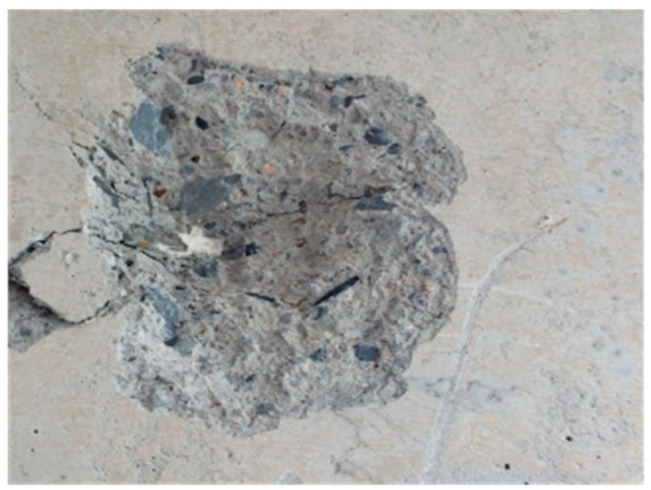
An example of concrete structure spalling.

In concrete structures, spalling is a serious issue that causes the exposure and potential corrosion of the reinforcing steel due to the removal of its protective concrete layer. Additionally, spalling is regarded as a significant indicator of severe damage to structural components during earthquakes. An example of spalling is illustrated in Figure 9. There have been a limited number of studies focused on the visual perception of spalling. German et al. [69] developed a spalling identification method that combines a global adaptive threshold algorithm with template matching and morphological operations, which is capable of measuring the depth and length of concrete column spalling. Dawood et al. [70] proposed a spalling detection method based on regression analysis and a hybrid algorithm, incorporating techniques such as image smoothing, thresholding, histogram equalization, Gaussian blur, color transformation, intelligent filtering, and image scaling. Furthermore, Gao et al. [71] developed a spalling classifier by retraining the VGG-16 network through deep transfer learning, aimed at identifying and categorizing spalling in concrete structures. Hoang et al. [72] introduced a machine learning method for classifying the severity of concrete spalling based on image texture analysis and a novel jellyfish search optimization. Nguyen et al. [73] suggested a method for classifying the severity of concrete spalling utilizing a meta-heuristic optimized extreme gradient boosting machine and a deep CNN.

Some scholars have recently researched methods for the perception of multiple types of defects. Huang et al. [74] proposed an automatic multi-damage detection method based on the improved Faster R-CNN, which is used to identify and locate different types of concrete dam damage. This method showed good effects regarding the detection of cracks, spalling, and sedimentation. Zhao et al. [75] proposed a system combining the You Only Look At Once V5s-HSC (YOLOv5s-HSC) algorithm and 3D photogrammetry reconstruction technology for the detection of concrete dam damages. This system incorporates Swin Transformer blocks and coordinate attention modules to enhance its feature extraction capabilities and employs a projection method to precisely locate and map the detected damages. Li et al. [76] utilized the lightweight YOLOv5 and Adaptive Spatial Feature Fusion (ASFF) technology to integrate input data enhancement, feature extraction, fusion, and multi-scale training processes, proposing a real-time automatic identification and quantification method for multiple defects in concrete dams. This method can effectively deal with defects such as dam cross-section cracks, water curtains, microcracks, and concrete spalling damages. Minh Dang et al. [77] proposed a transformer-based concrete defect identification model for the perception of four types of dam concrete defects. Hong et al. [78] have introduced a robotic solution for the automated detection, counting, and reconstruction of surface defects in dam spillways. This innovative approach merges deep learning methodologies with visual 3D reconstruction techniques, tackling challenges arising from limited real-world dam defect datasets and incomplete registration of minor defects during 3D mesh model fusion. It can be seen, from these studies, that interest in studying multi-type defect perception methods for high dam inspections has been increasing among scholars.

(2) Underwater Concrete Defect Detection

For submerged concrete structures, underwater concrete testing is imperative in high dam inspections. Underwater concrete structures are vulnerable to environmental influences like water quality and hydrostatic pressure, potentially resulting in diminished concrete integrity, crack propagation, and reinforcement corrosion. Significant progress has been made in the detection of defects on the surface of concrete dams using image processing techniques [79]. Chen et al. [80] proposed an adaptive underwater dam surface edge detection algorithm based on multi-structure and multi-scale elements. According to the differences between cracks and background regions in pre-processed images, crack detection algorithms based on local or global features have also been used to detect underwater cracks on dam surfaces. Fan et al. [81] applied prior knowledge obtained from the source domain to underwater crack image segmentation by using a multi-level adversarial transfer network to reduce the data labeling effort and integrated an attention mechanism into the segmentation network to achieve higher segmentation accuracy. Li et al. [82] researched a lightweight semantic segmentation and transfer learning method for pixel-level identification and quantification of underwater dam cracks and developed visualization software with offline and online functionalities. Furthermore, Qi et al. [83] introduced a three-step method for the automatic detection of micro-cracks in concrete during underwater structure operations. This method combines traditional approaches with deep learning techniques to accurately locate the cracks. Xin et al. [84] proposed a precise algorithm for identifying underwater surface cracks in dams from collected images. It employs adaptive histogram equalization to address the problem of uneven illumination, cluster analysis to extract crack areas, and Gaussian mixed models for classification. Their experimental results demonstrated a 90.1% extraction accuracy with low error rates. The merits and performance of each method are listed in Table 7.

### 3.3. Visual Perception Methods for Defect Quantification

To assess the potential risks of structural defects, it is generally necessary to quantify characteristics such as the size, shape, and depth of the defects. Some of the existing defect quantification methods are detailed in the following. Ni et al. [85] proposed a crack width estimation method based on Zernike moment operators. Wang et al. [86] developed a method to automatically measure the crack width using binary crack images. Rezaiee Pajand et al. [87] introduced a concrete crack detection approach utilizing genetic algorithms and finite element modeling to optimize crack detection performance and conduct a non-linear analysis of two-dimensional crack features, ultimately determining the location and size of the cracks. Ref. [88] investigated a UAV-based machine vision method for bridge crack recognition and width quantification through hybrid feature learning, which achieved over 90% accuracy in quantifying real bridge crack widths. Zhang et al. [89] proposed a crack quantification framework based on voxel reconstruction and Bayesian data fusion, which can identify the geometric properties of entire cracks using a set of unordered inspection images. Chen et al. [90] introduced a dam surface crack detection network based on deep learning, primarily addressing the semantic segmentation issue of dam surface cracks. Ding et al. [91] developed an improved calibration method that establishes the full-field scale of UAV gimbal cameras, allowing the scale factor to be conveniently indexed at various measurement attitudes without the need for recalibration. Additionally, an independent boundary refinement transformer (IBR-Former) was proposed for crack segmentation from UAV-captured images, wherein the IBR scheme can further refine the crack boundary in a model-agnostic manner. The proposed framework can quantify cracks with widths less than 0.2 mm.

The merits and performance of each method are listed in Table 7. Defect quantification provides vital data support for safety evaluations, and further research is warranted to enhance the precision and adaptability of these methods. The merits and performance of each method are listed in Table 8.

**Figure 10:**
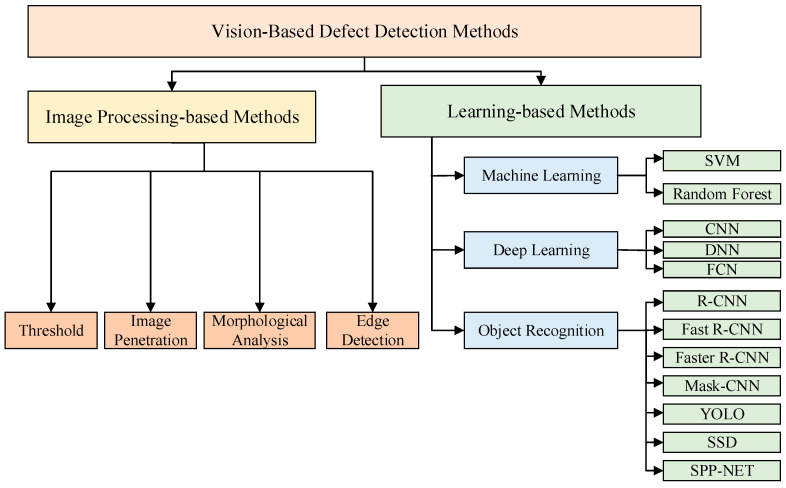
Classification of visual defect perception methods.

The classification of methods for the visual perception of concrete defects is depicted in Figure 10. The developmental trajectory has progressed from methods based on image processing and identification to those involving machine learning, deep learning, and object recognition. Due to insufficient research on the mechanisms and development patterns of structural damage and defects in high dams, along with a lack of in-depth analysis of damage characteristics at different stages, the designed perception methods often have limitations. Additionally, due to complex environmental factors such as low light, distortion, and sedimentation, the accuracy of defect localization is insufficient. The degree of automation in perception remains low, and the detection accuracy requires further improvement. Therefore, there are still many tasks to be undertaken to enable the effective identification and quantification of structural damage and defects in high dams.

## 4. Environmental Visual Perception Methods for High Dam Safety Inspection

In the task of intelligent safety inspections of high dams, methods allowing for the perception and understanding of unknown target entities in the complex inspection environment are key technologies ensuring the completion of adaptive inspection tasks. Due to the reliable and excellent perception capabilities of visual perception methods, they can obtain intuitive obstacle information from the inspection environment, providing data for the adaptive path planning of inspection equipment and ensuring the completion of inspection tasks.

This section reviews the development path of—and recent research on—obstacle visual perception methods for use in complex inspection environments. The background for obstacle visual perception in the environment of high dam safety inspections is static. Methods for obstacle perception against a static background can be divided into traditional image detection methods and deep learning-based target detection methods.

### 4.1. Obstacle Perception Methods Based on Traditional Image Detection

The traditional methods for image detection are classified into frame difference methods and texture feature methods. Mukojima et al. [92] proposed a background subtraction method that is suitable for mobile cameras, which calculates the frame correspondence between the current and reference image sequences, detecting obstacles through image subtraction of corresponding frames. Regarding texture feature-based detection methods, Tastimur et al. [93] applied HSV color transformation, image difference extraction, gradient computation, filtering, and feature extraction for object detection. This method employs a single camera to calculate the distance between the obstacle and the camera. Selver et al. [94] presented a robust approach that divides video frames into four regions, with each region being processed through wavelet filtering at different scales to enhance trajectory edges and filter noise. Teng et al. [95] utilized gradient histograms as extracted features and employed SVM as the learning algorithm to extract features from the superpixels of obstacles. Classic methods for image feature extraction include the Deformable Part Model (DPM) [96], Scale-Invariant Feature Transform (SIFT) [97], and Speeded Up Robust Features (SURFs) [98]. These methods extract image gradients and edge features for post-processing, offering stable detection performance; however, this comes at the cost of high time consumption and low robustness, thus failing to meet real-time requirements.

### 4.2. Obstacle Perception Methods Based on Deep Learning

In recent years, obstacle detection methods based on deep learning have been extensively applied. He et al. [99] proposed a rail traffic obstacle detection method based on an improved CNN. Addressing small object detection and multi-viewpoint scenarios, Li et al. [100] presented a cross-layer fusion multi-object detection and recognition algorithm based on Faster R-CNN, using a five-layer VGG16 structure to gather more feature information. As network structures have deepened and new frameworks have emerged, several new obstacle perception methods have been proposed. He et al. [101] enhanced the Mask R-CNN model to improve the augmentation and fusion of information at different scales, enhancing the feature extraction and fusion performance. Xu et al. [102] explored a method for the rapid detection of dynamic obstacles around moving agricultural machinery using a panoramic camera, employing optical flow algorithms to detect moving obstacles in panoramic images. Furthermore, Xue et al. [103] developed a farmland obstacle detection approach based on the improved YOLOv5s algorithm with CIOU and anchor box scale clustering. Yasin et al. [104] investigated a night vision obstacle detection and avoidance method based on bio-inspired visual sensors. Qiu et al. [105] proposed a method for the visual detection and tracking of moving obstacles in paddy fields based on improved YOLOv3 and Deep SORT, achieving obstacle detection and localization. In addition, Lalak et al. [106] studied multi-obstacle detection for UAVs based on monocular vision. Chen et al. [107] introduced a real-time recognition and avoidance method for both static and dynamic obstacles in UAV navigation point clouds, demonstrating certain advantages in tracking robustness, energy cost, and computation time. Chang et al. [108] suggested a spatial attention fusion obstacle detection method based on millimeter-wave radar and visual sensors, offering a novel approach that integrates visual methods with other sensors for obstacle recognition. The merits and limitations of each method are listed in Table 9.

**Figure 11:**
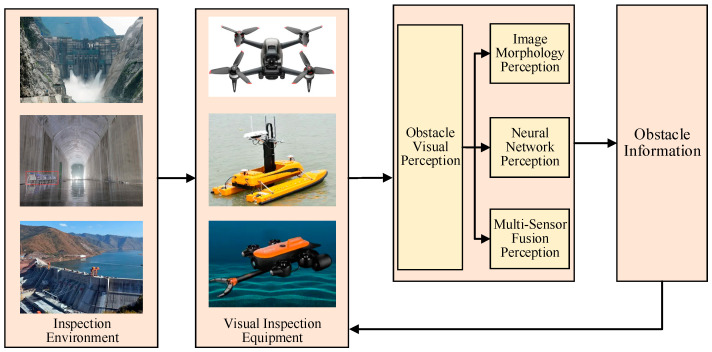
The technology path of intelligent inspection obstacle perception.

In summary, obstacle identification methods are gradually evolving towards the use of artificial intelligence techniques such as neural networks for object recognition. However, these methods still require comprehensive improvements to adapt to the complex tasks associated with safety inspections at high dams, considering the specific tasks and inspection carrier control methods. As shown in Figure 11, developing highly adaptable obstacle perception methods for inspection environments, based on the analysis of intelligent inspection theories and technologies in line with the safety requirements of high dams, is a worthwhile avenue for further research.

## 5. Conclusions

### 5.1. Summary

In this article, we provided a literature review on visual perception technologies for the intelligent inspection of high dam hubs. We first outlined the context and requirements of intelligent inspections in high dams, clarified the means of application of visual perception technologies, and analyzed the process of visual defect perception during high dam inspections. We also analyzed the characteristics of inspection images captured at high dams and detailed the research outcomes relating to image enhancement methods from the perspectives of traditional, Retinex theory, and deep learning approaches, outlining future directions for the development of image enhancement technologies tailored to the needs of high dam inspections.

We conducted a detailed analysis of the current state of research regarding visual perception methods for concrete surface defects in high dams. Traditional image processing methods primarily rely on preprocessing techniques, filtering methods, and thresholding to improve defect detection and recognition capabilities. While these methods have shown some effectiveness in specific scenarios, they are sensitive to threshold selection and have limited adaptability. Researchers have shifted their focus towards machine learning-based methods, which utilize support vector machines, clustering, image filtering, and preprocessing techniques to provide improved detection accuracy and robustness. However, these methods still require manual feature design and heavily depend on the empirical rules of the machine learning algorithms, posing challenges in extracting and recognizing diverse defect features. With the rapid development of deep learning, researchers have dedicated efforts to developing deep learning-based concrete surface defect perception methods. Convolutional Neural Networks (CNNs), VGG16, Faster-RCNN, Single Shot Detector (SSD), ResNet, and other deep learning network models, along with their improved variants, have been applied to study the perception of concrete surface defect types. The recent literature has focused on utilizing state-of-the-art network models such as YOLO series and Visual Transformer models, which have good feature recognition and context attention abilities, thereby achieving higher accuracy compared to existing models. Deep learning-based semantic segmentation algorithms have been widely used for concrete defect morphology perception, with various deep learning architectures being employed to improve the precision and efficiency in this context. Some researchers have also attempted to characterize and quantify crack defects from a three-dimensional perspective. Existing research has predominantly focused on concrete surface crack defects, with increasing attention being paid to the detection of various types of defects.

In terms of environmental perception, multiple deep learning networks are being used for obstacle detection, and some scholars have begun to focus on the recognition and boundary differentiation of high dam structures.

In summary, the field of visual perception for high dam inspections has seen significant advancements—particularly in the areas of image enhancement, defect detection, and environmental perception—driven by the integration of traditional image processing techniques, machine learning approaches, and deep learning methodologies. Future research trends may include further improvements to model robustness, the reduction in computational resources required, and the development of automated inspection systems that are suitable for real-world deployment.

### 5.2. Outlook

From the above analysis, visual perception methods can address some of the issues encountered in high dam safety inspections. However, due to the significant limitations of traditional image processing methods and the high data and computational demands of machine learning-based approaches, many challenges and opportunities still exist regarding the utilization of visual perception technology more effectively for intelligent safety inspections in both research and industrial applications.

1. Conducting intelligent inspection tasks through visual collection systems poses challenges related to factors such as complex objects, harsh environments, heavy inspection workloads, and a high incidence of emergent inspection tasks. The data captured by visual sensors on the inspection equipment may exhibit issues such as unclear images, jitter, and distortion. Systematically analyzing and semantically interpreting the complex inspection environment, and developing a highly adaptive collection system along with appropriate data preprocessing methods, are directions worthy of research.
2. Long-term inspections generate a large volume of image data, presenting challenges regarding data management, analysis, optimization, and visualization. Thus, there is an urgent need for research into the 2D and 3D visualization of inspection data, intelligent analysis, and high dam health assessment and risk prediction based on visual perception.
3. For the identification and quantification of structural defects, it is urgent to address issues such as efficient and precise parallel identification, localization, quantification, and 3D perception. The existing methods have limited defect sample data and low robustness. Future research can focus on developing visual perception methods based on small-sample/zero-sample learning, in order to solve the problem of limited defect data; undertaking multi-task learning method research to extract valuable information from multiple related tasks, in order to enhance the generalization ability of the designed algorithms; carrying out studies on multi-source heterogeneous data fusion combining radar, vibration, laser, and other sensors with visual sensors, in order to improve identification accuracy and efficiency of inspections; and exploring methods combining visual perception with structural modal analysis, in order to enhance the confidence level in safety evaluations.
4. In the task of intelligent patrol inspections for the safety of high dams, efficiently and accurately perceiving the complex inspection environment remains a challenge. To combine the advantages of existing methods with semantically enriched on-site environments, exploring multi-sensor fusion perception methods is a viable technical approach.

## Figures and Tables

**Table 1:** Comparison of histogram equalization methods for image enhancement.

Category	Method	Purpose	Merits
Histogram Equalization	AHE [11]	Adaptive histogram equalization algorithm	Enhances the local contrast and details of the image
	AHE [12]	Calculate local histograms based on the AHE method	Enhances image contrast while preserving a significant amount of detail
	WTHE [13]	Video enhancement based on the WTHE Algorithm	Over-enhancement and level saturation artifacts are effectively avoided
	LDR-HE [14]	Enhances images efficiently in terms of both objective and subjective quality	A novel contrast enhancement algorithm using the LDR of Lee et al.
	Underwater HE [15]	Enhances images efficiently in terms of both objective and subjective quality	Produces a pair of output versions

**Table 2:** Comparison of Retinex methods for image enhancement.

Category	Method	Purpose	Merits
Retinex	Retinex [16]	Remove or reduce the effects of illumination and preserve the essential characteristics of the object	Offers a robust and flexible framework for image enhancement tasks
	SSR [17]	Decompose an image into two different components: the reflection component and the illumination component	Reduces the effects of illumination and preserves the essential features of objects
	MSR [18]	Estimate the illumination component by combining several different scales using a central surround function	Balances local and global dynamic range compression
	SSRBF [19]	Address low and uneven lighting issues	Merges SSR and Bilateral Filter
	Retinex + HSV [20]	Achieve the goal of eliminating halo artifacts	Improves visibility and eliminates color distortion in HSV space
	RCF + CFAHE + NLF + GF [21]	Estimate illumination in the presence of spatially variant phenomena	Increases contrast, eliminates noise, and enhances details at the same time
	Fractional-order variational Retinex [22]	Enhance images with severely low light	Controls the regularization extent more flexibly
	Robust Retinex Model [23]	Address the issue that Retinex models often fail to deal with noise	Adaptable to a variety of tasks
	Concrete image enhancement [24]	Enhance the poor image quality of underwater concrete	Provides balanced enhanced images in terms of color, contrast, and brightness

**Table 3:** Summary of performance metrics of deep learning methods for image enhancement.

Method	Network	Dataset	PSNR	SIMM
LLnet [25]	Deep Autoencoder	Synthetic images	19.81	0.67
Hybrid network [26]	RNN	Synthetic images	28.43	0.96
Denoise [27]	CNN	Real images		0.85
CLAR [28]	Retinex + CNN	VV	19.65	0.61
DSICE [29]	CNN	Real under-exposed images	20.27	0.94
LightenNet [30]	CNN	Self	21.71	0.93
Retinex-Net [31]	CNN	LOL		
Retinex-GAN [32]	GAN	LOL	31.33	0.88
Global-SRA-U-net [33]	GAN	LOL	19.46	0.75
Deep Photo Enhancer [34]	GAN	MIT-Adobe 5K	23.80	0.90
EnlightenGAN [35]	GAN	LOL		
Weber-Fechner law in log space [36]	CNN	LOL	20.508	0.952
GPANet [37]	CNN	LOL	20.862	0.7842
LSR [38]	CNN	LOL	20.712	0.821
MFEF [39]	CNN	UIEB	23.352	0.910
LLFormer [40]	Transformer	LOL	23.6491	0.8163
Retinexformer [41]	Transformer	LOL	25.16	0.845

**Table 4:** The merits and limitations of crack detection methods based on traditional image processing approaches.

Reference	Method	Merits	Limitations
Fujita et al. [42]	Threshold	Handles irregular lighting conditions, shadows, and imperfections	Sensitivity and adaptability can be easily disrupted.
Fujita et al. [43]	Adaptive thresholds	Robust automatic crack detection from noisy concrete surface images	Suitable adaptive parameters and ranges must be selected
Talab et al. [44]	Otsu	Classifies background and foreground	Limited adaptation to images with complex background
Asdruabali et al. [45]	Threshold	Thermal image enhancement with Kantorovich operator	Adapted to infrared images
Chen et al. [46]	Otsu	Difference image adaptation for defect detection in complex background	The accuracy of noisy image processing needs to be further studied

**Table 5:** The merits and limitations of crack detection methods based on machine learning.

Reference	Method	Merits	Limitations
Liu et al. [47]	SVM	Utilizes the balanced local image	Limited accuracy
Luo et al. [48]	SVM	Adaptive binarization procedure	Black thin long writing and dirtiness are recognized as cracks.
Fisher et al. [49]	SVM	A novel data-driven approach	Generalization ability needs to be improved
Fan et al. [50]	Clustering	The threshold for realizing image binarization is self-adaptive	Limited environmental adaptability
Nishikawa et al. [51]	Genetic Encoding	Wavelet transforms at different scales	No scale adaptation
Gordan et al. [52]	Clustering	Fuzzy C-means clustering edge detection operator algorithm	High false alarm rate

**Table 6:** The merits and performance of crack detection methods based on deep learning.

Reference	Method	Merits	Dataset	Performance (%)
Dung et al. [53]	FCN	Reasonably detected and crack density is also accurately evaluated	Concrete Crack Images for Classification	AP = 89.3%
Ni et al. [54]	CNN	Delineates cracks accurately and rapidly	Own collection	Precision = 79.28%
Feng et al. [55,56,57]	CNN	Provides accuracy considerably higher than that of a support vector machine	Own collection	Precision = 93.48%
Modarres et al. [58]	CNN	Outperforms several other machine learning algorithms	Own collection	Accuracy = 99.6% Precision = 97.5%
Pang et al. [59]	RCNN	Crack segmentation method of hydro-junction project	Own collection	Iou = 52.7%
Li et al. [60]	CNN	Ideal for integration within intelligent autonomous inspection systems	Own collection	Accuracy = 80.7%
Deng et al. [61]	CNN	Defect detection on dam surface by using UAV tilt photogrammetry technology combined with machine vision	Own collection	Accuracy = 76.39%
Chen et al. [62]	FCN	Accurate identification and quantification of cracks on the dam surface	Own collection	Accuracy = 75.13%
Zou et al. [63]	U-Net	A novel end-to-end trainable convolutional network—DeepCrack	CRKWH100	AP = 93.15%
Li et al. [64]	Transfer Learning	Realized high-precision crack identification	Own collection	Precision = 91.23%
Zhu et al. [65]	Deeplab V3+	The fusion of a lightweight backbone network and attention mechanism	Own collection	Precision = 91.23%
Wu et al. [66]	LCA-YOLOv8-Seg	Suitable for low-performance devices	Concrete Crack Images for Classification	mAP = 93.30%
Zhang et al. [67]	UTCD-Net	Demonstrated superior generalizability with respect to complex scenes	CFD dataset	Precision = 62.85%
Xiang et al. [68]	DTrC-Net	More adaptable and robust to crack images captured under complex conditions	Crack3238	Precision = 75.60%

**Table 7:** The merits and performance of other defect detection methods.

Reference	Defect Type	Merits	Dataset	Performance (%)
German et al. [69]	Spalling	Automatically detecting spalled regions on a concrete structural element and retrieving significant properties of these spalled regions in terms of their length and depth	Own collection	AP = 80.90%
Dawood et al. [70]	Spalling	An integrated framework to detect and quantify spalling distress based on image data processing and machine learning	Own collection	Precision = 94.80%
Gao et al. [71]	Spalling	Spalling classifier based on deep transfer learning + VGGNet	Own collection	Accuracy = 91.50%
Hoang et al. [72]	Spalling	Image texture analysis and novel jellyfish search optimization method	Own collection	Precision = 93.20%
Nguyen et al. [73]	Spalling	Combination of meta-heuristic optimized extreme gradient boosting	Own collection	Precision = 99.03%
Huang et al. [74]	Multiple Types	An automatic multiple-damage detection method for concrete dams based on faster re-gion-based CNN	Own collection	mAP = 88.77%
Zhao et al. [75]	Multiple Types	A system for detecting damages in concrete dams that combines the proposed YOLOv5s-HSC algorithm and a three-dimensional (3D) photogrammetric reconstruction method to accurately identify and locate objects.	Own collection	mAP = 79.80%
Minh Dang et al. [76]	Multiple Types	accurately distinguish different types of structural defects in concrete dams under the interference of environmental noises.	Own collection	mAP = 89.40%
Hong et al. [77]	Multiple Types	An end-to-end transformer-based model	large-scale dataset	mAP = 63.80%
Chen et al. [80]	Underwater	This method enables accurate and efficient detection and classification of underwater dam cracks in complex underwater environments	Own collection	/
Fan et al. [81]	Underwater	The proposed method achieves accurate segmentation of underwater dam crack images	Own collection	Precision = 47.74%
Li et al. [82]	Underwater	Lightweight semantic segmentation and transfer learning	Own collection	Precision = 91.51%
Qi et al. [83]	Underwater	Combination of traditional approaches and deep learning techniques	Own collection	Accuracy = 93.9%
Xin et al. [84]	Underwater	Edge detection model based on artificial bee colony algorithm	Own collection	Precision = 90.10%

**Table 8:** The merits and performance of defect quantification methods.

Reference	Defect Type	Merits	Dataset	Performance (%)
Ni et al. [85]	Crack	The Zernike moment operator (ZMO) for achieving subpixel accuracy in measuring thin-crack width	Own collection	Precision = 88.65%
Wang et al. [86]	Crack	A new crack width definition and formulates it using Laplace’s Equation	Own collection	/
Rezaiee-Pajand et al. [87]	Crack	Concrete crack detection based on genetic algorithm	Own collection	Precision = 94.80%
Peng et al. [88]	Crack	crack recognition and width quantification through hybrid feature learning	Own collection	Precision = 92.00%
Zhang et al. [89]	Crack	Combination of voxel reconstruction and Bayesian data fusion	Own collection	AP = 87.3%
Chen et al. [90]	Crack	Combining semantic segmentation and morphology	Own collection	Precision = 90.81%
Ding et al. [91]	Crack	Based on IBR-Former, it can quantify cracks with widths less than 0.2 mm	Own collection	Precision = 85.32%

**Table 9:** The merits and limitations of obstacle perception methods.

Perception Type	Reference	Method	Merits	Limitations
Traditional Image Detection	Mukojima et al. [92]	background subtraction algorithm	Moving camera background subtraction for forward obstacle detection	Limited light adaptation
	Tastimur et al. [93]	background subtraction algorithm	HSV color transformation, image difference extraction, gradient computation, filtering, and feature extraction	Foreign objects in the level crossing are not enough
	Selver et al. [94]	Gabor wavelets	Trajectory edge enhancement and noise filter	Limited detection range
	Teng et al. [95]	background subtraction algorithm	Combination of multiple sensors and the vision-based snag detection algorithm	Limited detection range
Deep Learning	He et al. [99]	FE-YOLO	A flexible and efficient multiscale one-stage object detector	Limited real-time performance
	Li et al. [100]	Faster R-CNN	Multi-object detection and recognition	Limited generalization ability
	He et al. [101]	Mask R-CNN	High precision in small target detection	Limited generalization ability
	Xu et al. [102]	Optical flow algorithm	Dynamic obstacle detection based on panoramic vision	Single direction of motion
	Xue et al. [103]	Improved YOLOv5s	The small-weight file	Limited generalization ability
	Yasin et al. [104]	Hough transform	Used an adaptive slicing algorithm based on accumulating number of events	Limitation to specific scenarios
	Qiu et al. [105]	YOLOv3	Vision-based moving obstacle detection and tracking	No center point detection
	She et al. [106]	YOLOv3 + SURF	Multi-obstacle detection	Limited generalization ability
	Chen et al. [107]	The forbidden pyramids	Real-time identification and avoidance of simultaneous static and dynamic obstacles	Limited robustness
